# Deep learning *in vivo* catheter tip locations for photoacoustic-guided cardiac interventions

**DOI:** 10.1117/1.JBO.29.S1.S11505

**Published:** 2023-11-13

**Authors:** Mardava R. Gubbi, Fabrizio Assis, Jonathan Chrispin, Muyinatu A. Lediju Bell

**Affiliations:** a Johns Hopkins University, Department of Electrical and Computer Engineering, Baltimore, Maryland, United States; b Johns Hopkins Medical Institutions, Division of Cardiology, Baltimore, Maryland, United States; c Johns Hopkins University, Department of Biomedical Engineering, Baltimore, Maryland, United States; d Johns Hopkins University, Department of Computer Science, Baltimore, Maryland, United States

**Keywords:** photoacoustics, imaging, deep learning, detection, computer vision, phased arrays

## Abstract

**Significance:**

Interventional cardiac procedures often require ionizing radiation to guide cardiac catheters to the heart. To reduce the associated risks of ionizing radiation, photoacoustic imaging can potentially be combined with robotic visual servoing, with initial demonstrations requiring segmentation of catheter tips. However, typical segmentation algorithms applied to conventional image formation methods are susceptible to problematic reflection artifacts, which compromise the required detectability and localization of the catheter tip.

**Aim:**

We describe a convolutional neural network and the associated customizations required to successfully detect and localize *in vivo* photoacoustic signals from a catheter tip received by a phased array transducer, which is a common transducer for transthoracic cardiac imaging applications.

**Approach:**

We trained a network with simulated photoacoustic channel data to identify point sources, which appropriately model photoacoustic signals from the tip of an optical fiber inserted in a cardiac catheter. The network was validated with an independent simulated dataset, then tested on data from the tips of cardiac catheters housing optical fibers and inserted into *ex vivo* and *in vivo* swine hearts.

**Results:**

When validated with simulated data, the network achieved an F1 score of 98.3% and Euclidean errors (mean ± one standard deviation) of 1.02±0.84  mm for target depths of 20 to 100 mm. When tested on *ex vivo* and *in vivo* data, the network achieved F1 scores as large as 100.0%. In addition, for target depths of 40 to 90 mm in the *ex vivo* and *in vivo* data, up to 86.7% of axial and 100.0% of lateral position errors were lower than the axial and lateral resolution, respectively, of the phased array transducer.

**Conclusions:**

These results demonstrate the promise of the proposed method to identify photoacoustic sources in future interventional cardiology and cardiac electrophysiology applications.

## 1 Introduction

Cardiac interventional procedures are often performed to diagnose and treat cardiac arrhythmias (e.g., ∼18,000 to 44,500 cardiac catheter ablation procedures have been performed annually in the United States^1^). These procedures generally require catheter delivery from an insertion point in the thigh to the heart via the femoral vein. One of the most serious and potentially life-threatening complications of catheter ablations is the risk of cardiac perforation,^1^ which can be minimized with state-of-the-art catheter tip visualization methods.

A combination of fluoroscopy^2^^,^^3^ and intracardiac ultrasound^4^ is currently used to provide the real-time localization information of the catheter tip within the heart needed to mitigate complications and to guide the catheter tip toward targets of interest. However, fluoroscopy exposes both patients and operators to ionizing radiation,^5^^,^^6^ resulting in biological effects^7^ such as radiodermatitis,^8^^,^^9^ increased cancer risks,^10^^–^^13^ and genetic defects,^11^^,^^13^ from catheter ablation procedures requiring fluoroscopy.^2^ Additional challenges include the lack of depth information in monoplane fluoroscopic images, resulting in catheter tip depth localization errors of up to 10 mm,^14^ and the poor fluoroscopic contrast of anatomical features limiting catheter tip localization relative to surrounding anatomy.^3^ While intracardiac ultrasound imaging generally provides suitable views of a cardiac catheter, it does not provide depth information and it requires additional fluoroscopy, electromagnetic tracking, and skilled operators to provide a more global reference frame.^15^ Transthoracic ultrasound imaging is a potential option to provide depth information, but it is challenged by acoustic clutter,^16^ catheter tips having similar echogenicity to the myocardium,^17^ and shadowing from the ribs.^18^

Photoacoustic imaging coupled with robotic visual servoing was previously introduced as a method to guide biopsy needles in phantoms and *ex vivo* tissue samples^19^^,^^20^ and catheter tips *in vivo*.^17^^,^^21^ Photoacoustic imaging utilizes pulsed laser light to excite optical absorbers in a region of interest. These absorbers convert the absorbed optical energy to acoustic energy (i.e., mechanical pressure waves), which can be sensed by a standard ultrasound transducer, then reconstructed to create a photoacoustic image.^22^^–^^25^ When coupled with visual servoing, a robot arm holds the ultrasound transducer, and a dedicated algorithm segments the tip of the optical fiber in the beamformed image.^19^^,^^20^^,^^26^ The robot then tracks the fiber tip and guides the transducer to a desired location that centers the photoacoustic signal in the image. Therefore, photoacoustic visual servoing coupled with ultrasound imaging has the potential to overcome the limitations of existing catheter guidance techniques (e.g., fluoroscopy) by not requiring exposure to ionizing radiation, by providing depth information relative to a body surface, and by offering the global reference frame of the robot arm.^17^^,^^21^

Despite the many benefits of photoacoustic visual servoing coupled with ultrasound imaging, reflection artifacts resulting from highly echoic structures cause bright reflections in the beamformed photoacoustic image, which can be challenging for the segmentation step.^19^^,^^20^^,^^27^ To overcome this challenge, deep learning methods were previously leveraged to identify needle tips and catheter tips directly from raw photoacoustic channel data rather than beamformed images.^28^^–^^32^ In particular, a convolutional neural network was trained with simulated data to detect photoacoustic point sources,^28^^–^^32^ including photoacoustic signals originating from an optical fiber tip housed in either a needle surrounded by water,^29^^–^^31^ a needle surrounded by *ex vivo* tissue,^32^ or a cardiac catheter located in an *in vivo* femoral vein.^32^ This previous work also demonstrates the importance of correctly modeling the ultrasound receiver when implementing deep learning to detect photoacoustic sources and remove reflection artifacts.^30^ These major contributions were initially demonstrated with a linear array ultrasound transducer.^28^^–^^32^ Subsequent work from our group demonstrated the applicability of these techniques to detect needle tips and catheter tips in simulated and *in vivo* intravasular photoacoustic channel data acquired with phased array transducers.^33^^,^^34^ In cardiac imaging applications, phased array transducers are desirable due to their lower acoustic frequencies (which enable increased imaging depths), their smaller physical footprint when imaging between the ribs, and their larger image field of view (FOV) relative to their footprint.

In addition to developing a deep learning catheter tip detection method with a phased array transducer, we developed a deep learning-based photoacoustic visual servoing system using a phased array transducer.^35^ This system identified and tracked the tip of a hollow-core needle in a plastisol phantom and *ex vivo* chicken breast tissue, based on information provided in raw channel data, thus completely bypassing the image formation and segmentation steps. While this deep learning-based photoacoustic visual servoing system reduced needle tip tracking errors relative to a segmentation-based system,^35^ the required deep learning-based source detections were susceptible to misclassification errors when translated from the simulation to experimental domain, resulting in an increased reliance on temporal checks to verify the validity of the detected source position. These temporal validity checks were implemented across multiple consecutive frames, which reduces the maximum possible movement speed for successful *in vivo* tracking of the needle tip.

In this paper, we present our achievements when translating our deep learning approach from simulated data, plastisol phantom data, and *ex vivo* chicken breast tissue data to *ex vivo* and *in vivo* cardiac data, including new technical strategies when using the desired phased array transducer. We first train a network with simulated channel data frames, which are formatted to accommodate the FOV of a phased array transducer, including multiple noise levels, signal amplitudes, and sound speeds to ensure robustness to channel noise, target amplitude, and sound speed differences. We additionally introduce a new approach to improve network performance on *ex vivo* and *in vivo* data by matching the amplitude histograms of the experimentally acquired and simulated channel data frames. We validate our network on previously unseen simulated data and test our network on *ex vivo* and *in vivo* cardiac data. In addition, we characterize the performance of the trained network on the *ex vivo* and *in vivo* cardiac data before and after the histogram matching transformation to demonstrate the advantages of the transformation in the context of point source localization. While this successful source localization performance is sufficient for our deep learning-based photoacoustic visual servoing system, we also render network-based images of the detected simulated, *ex vivo*, and *in vivo* photoacoustic sources to qualitatively demonstrate the ability of our system to improve photoacoustic source visibility in cardiac applications.

The remainder of this paper is organized as follows. Section 2 describes the processes implemented for our simulation methods, experimental data acquisition, network training and testing, performance assessment, and visualization approaches. Section 3 details the results of the presented methods. Section 4 discusses the implications and future potential of our work, and Sec. 5 concludes the paper with a summary of our major findings.

## 2 Methods and Materials

### 2.1 Datasets

#### 2.1.1 Simulated datasets for training and validation

Channel data received by a phased array transducer were simulated in k-Wave.^36^ Each simulation consisted of a point source in a two-dimensional (2D) simulation grid consisting of a homogeneous medium. The top row of each simulation grid was populated with sensing elements to record the local pressure distribution at each time instant of the simulation. The initial pressure distribution corresponding to the point source was smoothed using a Blackman filter.^37^ The sensing elements were designed to simulate an Alpinion (Seoul, South Korea) SP1-5 phased array ultrasound transducer with an element width of 220  μm, a kerf of 80  μm, an aperture width of 19.2 mm, and a sampling frequency of 40 MHz. Each simulated channel data frame contained 3117 total samples in the axial dimension (i.e., 12 cm imaging depth with sound speed 1540  m/s), with additional simulation parameters describing the received channel data listed in Table 1.

**Table 1:** Range and increment size of simulated point targets and surrounding media.

Parameters	Min	Max	Increment
Axial position (mm)	20	100	0.25
Lateral position (mm)	−57	57	0.25
Channel SNR (dB)	−5	2	Random
Object intensity (multiplier)	0.75	1.1	Random
Speed of sound (m/s)	1440	1640	6

A total of 20,000 raw photoacoustic channel data frames were generated. Each frame contained a waveform corresponding to a point source of diameter 0.1 mm. In addition, a random subset of the frames contained an additional waveform corresponding to a reflection artifact. These reflection artifacts were generated as described by Allman et al.^31^ (i.e., a true photoacoustic source signal was shifted deeper into the image by the Euclidean distance between the source and reflector locations).

**Fig. 1:**
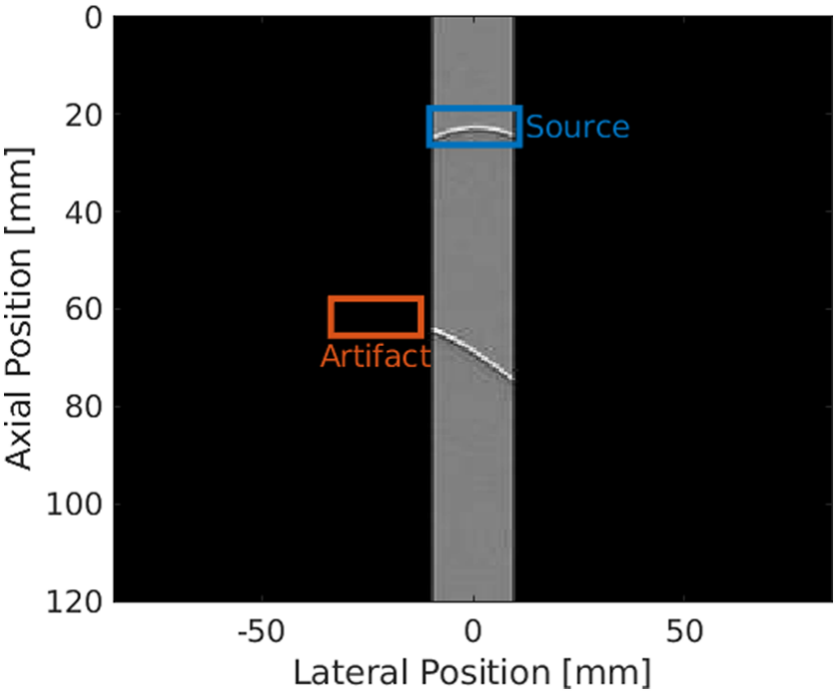
Example channel data image surrounded by zero-padded regions to match the dimensions of a beamformed, scan-converted image, including one source located directly under the transducer aperture and one reflection artifact with a wavefront peak located outside the transducer aperture. The locations of the peaks of the source and artifact wavefronts are denoted by the blue and orange bounding boxes, respectively.

Unlike implementations for a linear array transducer,^28^^–^^32^ the FOV of a phased array transducer in a scan-converted image extends laterally beyond the width of the raw channel data frame.^33^^–^^35^ To implement this additional constraint, the channel data frames were zero-padded to match the dimensions of a scan-converted phased array image, as demonstrated in Fig. 1. To improve the performance of the network^38^^,^^39^ and reduce the overall training and inference times,^40^ these zero-padded channel data frames were then resized from their original dimensions of 1132×3117  pixels to 256×256  pixels. This resizing increased the width and height of each pixel corresponding to the lateral and axial image dimensions, respectively (e.g., from 150.0 and 38.5  μm, respectively, to 662.9 and 468.8  μm, respectively, when the sound speed was 1540  m/s). For brevity, these zero-padded and resized channel data frames will be referred to as processed channel data frames.

For each processed channel data frame, bounding boxes of dimensions 32×16  pixels were generated, centered on the positions of sources and artifacts within the frame. These bounding boxes were allowed to exist in the zero-padded region, as shown in Fig. 1. The coordinates and class (i.e., source or artifact) of each bounding box are collectively referred to as position annotations. An annotated image in the simulated dataset consisted of the processed channel data frame combined with the corresponding position annotations. The totality of annotated images were randomly split into training (80%) and validation (20%) datasets.

#### 2.1.2 *Ex vivo* and *in vivo* datasets for testing

**Fig. 2:**
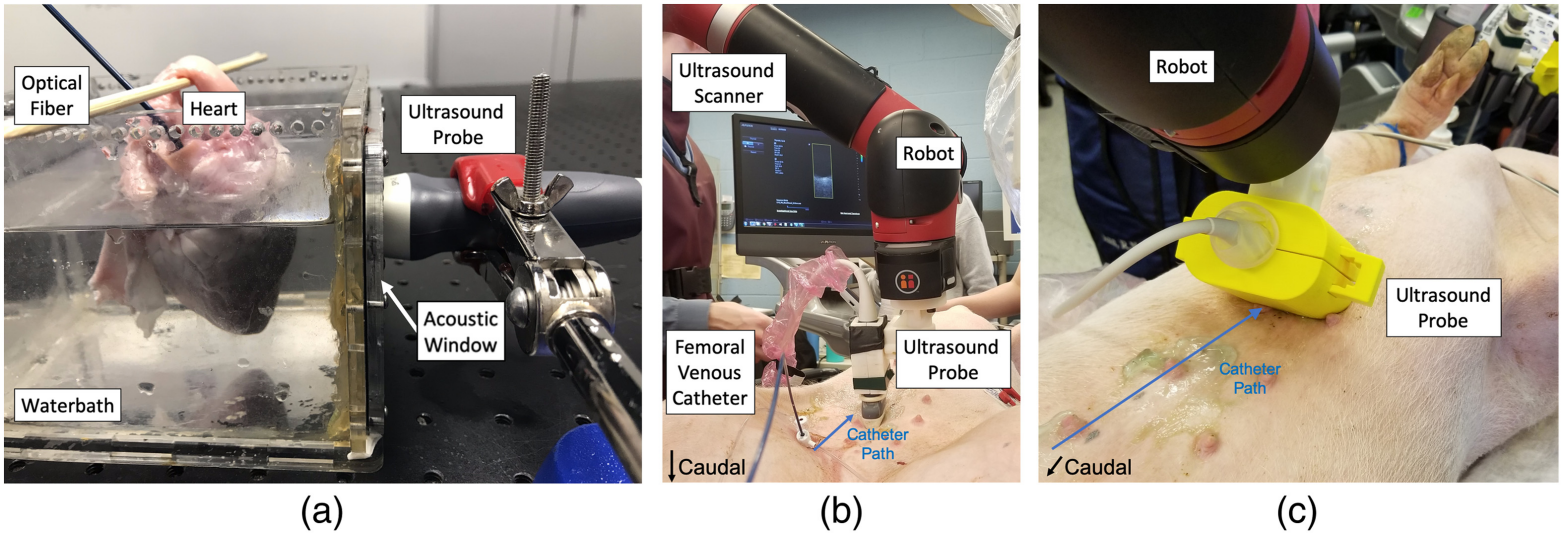
Experimental setups to acquire *ex vivo* and *in vivo* cardiac data. (a) The *ex vivo* setup contained a swine heart suspended in a waterbath with an optical fiber inserted into the inferior vena cava. The ultrasound transducer was placed in contact with the acoustic window of the box to perform imaging. The *in vivo* setup contained an ultrasound transducer attached to the end effector of a Sawyer robot and placed in contact with a swine. The ultrasound transducer was used to (b) track the trajectory of the tip of a catheter-fiber pair advanced via the femoral vein to the heart and (c) acquire channel data from the tip of the catheter–fiber pair located in the right atrium of the heart, with the transducer placed to obtain a subcostal view.

To acquire *ex vivo* experimental data, a swine heart was excised and suspended in a waterbath inside an acrylic box with an acoustic window on one side, as shown in Fig. 2(a). A 1 mm core-diameter optical fiber was inserted through the inferior vena cava into the right atrium and right ventricle. The other end of the optical fiber was coupled to a Phocus Mobile laser (Opotek, Carlsbad, California) operating at a 750 nm wavelength with a pulse rate of 10 Hz. The fiber tip was imaged by an Alpinion (Seoul, South Korea) E-Cube 12R scanner connected to an SP1-5 phased array ultrasound transducer. The ultrasound transducer was fixed to the acoustic window using a clamp. This photoacoustic imaging system was used to acquire 233 channel data frames at an imaging depth of 12 cm with varying transducer positions and optical fiber insertion depths.

To acquire *in vivo* data using the same photoacoustic imaging system described above, two swine were catheterized with approval from the Johns Hopkins University Animal Care and Use Committee. Each swine was fully anesthetized and positioned supine on an operating table. A 1 mm core-diameter optical fiber was inserted into a 5F inner-diameter cardiac catheter (St. Jude Medical, St. Paul, Minnesota) forming a fiber-catheter pair. The ultrasound transducer was held in place by a Sawyer Robot (Rethink Robotics, Boston, Massachusetts), as shown in Fig. 2(b), which overviews the entire *in vivo* setup. After the fiber-catheter pair was inserted in a femoral vein sheath and advanced toward the heart [Fig. 2(c)], the laser was pulsed at a wavelength of 750 nm and raw channel data frames were acquired with the catheter tip located in the heart while imaging at a depth of 12 cm. A total of 30 and 40 raw channel data frames were acquired during the first and second swine procedures, respectively, with average laser energies of 2.67 mJ and 608.5  μJ, respectively (corresponding to fluence values at the fiber tip of 340 and $19.37\;\mathrm{mJ}/{\mathrm{cm}}^{2}$, respectively). Data from the first and second *in vivo* experiments described herein were initially published by Graham et al.^17^ and Gonzalez et al.,^41^ respectively. As noted by Graham et al.,^17^ the laser fluence of $340\;\mathrm{mJ}/{\mathrm{cm}}^{2}$ during the first *in vivo* experiment exceeded the $25.6\;\mathrm{mJ}/{\mathrm{cm}}^{2}$ safety limit defined by the American National Standards Institute for human skin at a wavelength of 750 nm.^42^ However, no safety limits are currently defined for lasers in direct contact with cardiac tissue, and a histopathological analysis of the excised swine heart revealed no pathologic changes.^17^

For each channel data frame acquired during the *ex vivo* and *in vivo* experiments, a photoacoustic image was reconstructed using delay-and-sum (DAS) beamforming, then scan converted to manually identify the position of the fiber tip in the image and generate the corresponding position annotations for the image. Each raw channel data frame was also zero-padded and resized to form a processed channel data frame, which was then combined with the corresponding position annotations to form an annotated image. Hereafter, the set of *ex vivo* annotated images will be referred to as the “*Ex Vivo* Heart” dataset, and the sets of *in vivo* annotated images from the first and second swine catheterization procedures will be referred to as the “*In Vivo* Heart 1” and “*In Vivo* Heart 2” datasets, respectively.

### 2.2 Network Architecture and Training Procedure

A Faster R-CNN network^43^ with a Resnet-101^44^ feature extractor was implemented to determine point source locations. This network was initialized with pre-trained weights from the ImageNet dataset,^45^ then fine-tuned for 20 epochs with a batch size of 4 and a base learning rate of $1\times 10^{-3}$ on two NVidia (Santa Clara, California) Titan X (Pascal) GPUs, using data parallelization and the gradient aggregation method described by Goyal et al.^46^ This fine-tuning process was performed using the training dataset described in Sec. 2.1.1 and the Detectron2 software package.^47^ The network was trained to detect each acoustic waveform present in an input channel data frame, classify the waveform as corresponding to a point source or reflection artifact, and locate the peak of the detected waveform. This peak was not visible in the photoacoustic channel data when the lateral location of the source or artifact resided in the zero-padded region, as shown in Fig. 1. In this case, the network was required to both classify the waveform and extrapolate the position of its peak using the visible portion of the waveform present in the input channel data frame. The network outputs for each input image were formatted as a list of object detections consisting of the identified class (i.e., source or artifact), the object location (i.e., bounding box pixel coordinates), and a confidence score between 0 and 1.

When implemented on the two NVidia GPUs noted above, the network training process took ∼4  h to complete. After training, the network performed inference on input images at an average rate of 0.074 s per image, translating to an achievable frame rate of 13.5 Hz for real-time photoacoustic source localization.

### 2.3 Validation and Testing

#### 2.3.1 Filtering based on confidence scores and evaluation of network detections

To evaluate the performance of our network on the validation dataset described in Sec. 2.1.1, we filtered the network detections based on their confidence scores using an optimal confidence score threshold described below, then defined the retained detections as true positives, false positives, or misclassifications using their bounding box coordinates and classes described in Sec. 2.2. A network detection was defined as a true positive based on three criteria: (1) the confidence score of the detection was above the optimal threshold, (2) a ground truth of the same class was present in the associated annotations, and (3) the intersect-over-union between the bounding boxes of the detection and the ground truth was greater than 0.5. A network detection was defined as a false positive if it satisfied criterion 1 above, but either failed criterion 2, or satisfied criterion 2 and failed criterion 3. A network detection was further categorized as a misclassification if it met the definition of false positives above and, in addition, there was a ground truth of the opposite class (i.e., source ground truths for artifact detections and vice versa) satisfying criterion 3 above. Detections with confidence scores greater than the optimal threshold corresponding to their class were retained, and the remaining detections were discarded. These retained detections and their definitions (i.e., true positive, false positive, or misclassification) were then used to compute the recall, precision, and F1 scores,^48^ as well as the misclassification and missed detection rates^31^ for the source and artifact classes in the validation dataset.

To compute the optimal confidence score thresholds, we utilized the technique presented by Allman et al.^31^ using the corresponding receiver operating characteristics (ROC) curves for each class (i.e., source or artifact). These ROC curves represented the quality of network detections and were characterized using the area under the curve (AUC) reported separately for each class.^49^^,^^50^ To construct the ROC curve for each class, we varied the confidence score threshold from 0 to 1, filtered the network detections in the validation dataset based on the confidence scores, then computed the true positive rate and false positive rate using the definitions of true positives and false positives above. Once the ROC curve was constructed, a line was defined with a slope equal to the number of false positives divided by the number of true positives for that class, assuming a confidence score threshold of zero. This line was then shifted from the ideal operating point (i.e., the point with a true positive rate of unity and a false positive rate of zero) down and to the right until it intersected with the ROC curve. The first intersection of this line with the ROC curve was determined to correspond to the optimal confidence score threshold for the given class (i.e., 0.526 and 0.719 for the source and artifact classes, respectively).

To evaluate the performance of the network on the test datasets described in Sec. 2.1.2, the network detections for each test dataset were filtered using the optimal confidence score thresholds computed for the validation dataset (i.e., 0.526 and 0.719 for the source and artifact classes, respectively), then categorized as true positives, false positives, or misclassifications based on the definitions above. Recall, precision, F1 score, misclassification rate, and missed detection rate (i.e., the same performance metrics reported for the validation dataset) were computed for the source class using the retained detections for each test dataset.

#### 2.3.2 Histogram matching to improve network performance

As described in Sec. 2.1.2, our experimental datasets were acquired with different laser energies and additional processing was applied to match these datasets to the dimensions and structure of the annotated images in the simulated datasets for testing purposes. These factors contributed to dissimilarities between our simulated and acquired datasets as well as information loss in the *ex vivo* and *in vivo* datasets, which adversely affected target detectability in the processed channel data frames. This reduction in target detectability is anticipated to limit the ability of our network to detect and localize targets in the *ex vivo* and *in vivo* processed channel data frames.

To improve the performance of the simulation-trained network on *ex vivo* and *in vivo* data, histogram matching was performed, using the simulated dataset as a reference. To implement histogram matching, amplitude histograms were created for each processed channel data frame described in Sec. 2.1, using the inclusive range 0 to 255 with 64 bins. Each processed channel data frame in the *ex vivo* and *in vivo* datasets (described in Sec. 2.1.2) was then transformed to match the reference histogram of a randomly selected processed channel data frame in the simulated dataset (described in Sec. 2.1.1), which we refer to as histogram-matched channel data frames.

#### 2.3.3 Quantifying effects of histogram matching on *ex vivo* and *in vivo* images

To quantify the impact of histogram matching on improving the similarity between *ex vivo* and *in vivo* processed images and the reference simulated processed images, we utilized the total variation distance (TVD, described as intersection distance by Cha^51^), the Jeffrey divergence^52^ (JD), and the $\chi^{2}$ statistic.^53^ Each processed channel data frame in the *ex vivo* and *in vivo* datasets was normalized, amplitude histograms were constructed, and the TVD, JD, and $\chi^{2}$ statistics were computed using the following expressions:

$$\mathrm{TVD}=1-\overset{255}{\underset{k=0}{\sum }}\;\min\{h_{e}(x_{k}),h_{s}(x_{k})\},$$ (1)

$$\mathrm{JD}=\overset{255}{\underset{k=0}{\sum }}[h_{e}(x_{k})\log(\frac{h_{e}(x_{k})}{h_{e}(x_{k})+h_{s}(x_{k})})+h_{s}(x_{k})\log(\frac{h_{s}(x_{k})}{h_{e}(x_{k})+h_{s}(x_{k})})],$$ (2)

$$\chi^{2}=\overset{255}{\underset{k=0}{\sum }}[\frac{{\left(h_{e}(x_{k})-h_{s}(x_{k})\right)}^{2}}{h_{e}(x_{k})+h_{s}(x_{k})}],$$ (3)

where $h_{e}$ and $h_{s}$ are the amplitude histograms constructed from pixels in corresponding experimental and reference (i.e., simulated) processed channel data frames, respectively (using the inclusive range 0 to 255 with 256 bins), and $x_{k}$ is the mean value of k’th bin. These metrics were additionally implemented with histogram-matched channel data frames replacing processed channel data frames to achieve the desired comparisons of pre- and post-histogram-matching results.

To quantify the effect of histogram matching on the detectability of photoacoustic point sources in the *ex vivo* and *in vivo* datasets, we utilized the generalized contrast-to-noise ratio (gCNR), a metric initially designed to measure target detectability in ultrasound images,^54^ with previously demonstrated applications to photoacoustic imaging.^55^^,^^56^ Although gCNR was previously measured after implementing beamforming in these cases, the same principle of separability between target and background regions is applicable to the recorded waveforms in photoacoustic channel data frames originating from point sources. Therefore, gCNR is uniquely utilized herein to provide information about the separability of waveform signals from their surrounding background in the channel data. To calculate this channel gCNR, target and background regions of interest (ROIs) of size 18.2 mm (width) × 5 mm (height) were first defined in the zero-padded channel data frames in the *ex vivo* and *in vivo* datasets, then copied to the same locations in corresponding processed and histogram-matched channel data frames originating from the same raw data. Each target ROI was laterally centered in the corresponding image and axially shifted 1 mm distal to the ground truth source position to surround the waveform corresponding to the point source. Each background ROI was located 10 mm proximal to the corresponding target ROI to ensure complete separation between the two ROIs. After normalizing each image to the brightest pixel and extracting pixel amplitudes from the target and background ROIs, power histograms $h_{i}$ and $h_{o}$ were constructed for the target and background regions, respectively, using the inclusive range 0 to 1 with 256 bins, and channel gCNR was measured from these histograms as follows:

$${\mathrm{gCNR}}_{\mathrm{ch}}=1-\overset{255}{\underset{k=0}{\sum }}\;\min\{h_{i}(x_{k}),h_{o}(x_{k})\},$$ (4)

where $h_{i}$ and $h_{o}$ were derived from zero-padded, processed, or histogram-matched channel data frames in the *ex vivo* and *in vivo* datasets.

To determine the impact of histogram matching on the final outputs of interest, the histogram-matched channel data frames from the *ex vivo* and *in vivo* datasets were input to the trained network, and the detections output by the network were filtered and categorized as true positives, false positives, or misclassifications using the procedure for test datasets described in Sec. 2.3.1. The associated performance metrics (i.e., recall, precision, F1 score, misclassification rate, and missed detection rate) were computed for the source class. These metrics were then compared to the metrics obtained with processed channel data frames prior to histogram matching.

### 2.4 Source Localization Performance Metrics

To establish a baseline for the source localization performance achievable by our network, we measured the lateral and axial resolution of our photoacoustic imaging system with a 450  μm-diameter copper wire suspended in a water bath and illuminated by a 5 mm-diameter optical fiber bundle. Note that the diameter of this wire is considered to be consistent with that of a point target, because it is smaller than the theoretical resolution of our imaging system (i.e., the wire is a line target).^17^^,^^57^^,^^58^ The opposite end of the fiber bundle was interfaced to the Phocus Mobile laser described in Sec. 2.1.2. The illuminated portion of the wire was imaged using the Alpinion E-Cube 12R scanner and SP1-5 transducer mentioned in Sec. 2.1.2. The transducer was affixed to a UR5e (Universal Robots, Denmark) robotic arm. Photoacoustic channel data were acquired with the wire laterally centered underneath the transducer (i.e., lateral position of 0 mm) to match the lateral positions of the majority of targets in the *ex vivo* and *in vivo* datasets. The axial position of the wire was varied by moving the robot arm in 10 mm increments, resulting in axial target depths spanning 20 to 100 mm, which is similar to the ranges of axial positions occurring in the simulated (i.e., 20 to 100 mm), *ex vivo* (i.e., 43.17 to 63.23 mm), and *in vivo* datasets (i.e., 63.03 to 91.62 mm). At each fixed position of the wire, 50 frames of raw channel data were acquired. Photoacoustic images were reconstructed from these channel data frames using DAS beamforming, and the resolution was measured as the full width at half maximum^57^^,^^58^ of the target in the lateral and axial dimensions of each beamformed image.

To quantify the source localization accuracy of our network, we implemented two distinct processes for the simulated and *ex vivo* or *in vivo* datasets. For the simulated training and validation datasets, the absolute lateral, absolute axial, and Euclidean distance errors between the ground truth and detected sources were measured as functions of the ground truth source positions in the annotated image. The mean ± one standard deviation of the position errors was reported for each simulated dataset. In addition, these errors were reported separately for ground truth positions directly underneath and outside the transducer aperture to demonstrate the difference in localization performance when the wavefront peak was either visible or not visible in the channel data region. Finally, the absolute lateral and axial position errors were reported separately for ground truth axial positions in the range 15 to 105 mm, separated into nine distinct groups (for direct comparison with resolution measurements, which were obtained in 10 mm increments, as described above). To form these nine groups, position errors were sorted based on the associated ground truth positions, with ground truth axial positions greater than an odd multiple of 5 mm and less than or equal to the next odd multiple of 5 mm included in the same group (e.g., group 1 is defined by errors associated with: 15 mm < ground truth axial positions ≤25  mm). Similarly, the absolute lateral and axial position errors were reported separately for ground truth lateral positions, incremented by 10 mm for comparison with the axial position groupings.

For each *ex vivo* and *in vivo* test dataset, we reported lateral and axial position errors between the network detections and manually annotated ground truth source positions. These results are not further split into lateral regions as implemented for the simulated data because a majority of these data were acquired with the catheter tip directly underneath the transducer in the lateral dimension. However, the lateral and axial position errors were reported separately for ground truth axial positions in the range 35 to 95 mm, separated into six distinct groups incremented by 10 mm, for direct comparison with the resolution measurements, as described above.

### 2.5 Visualizing Sources Using Network Position Estimates

To demonstrate the potential for visual display of the phased array network outputs, we employed the artifact removal method proposed by Allman et al.^31^ Examples of estimated source positions from each dataset were each represented within a grid matching the FOV of a DAS-beamformed and scan-converted image. Each source was plotted as a circle centered on the estimated source position with radius of 2σ, where σ is the standard deviation of the Euclidean distance errors in the simulated validation dataset. These network-based images were visually compared with images reconstructed using traditional DAS beamforming and scan conversion to demonstrate the improved source visibility and the absence of reflection artifacts in the network-based images. The generation of human-interpretable images with improved source visibility is one alternative application of the outputs of a deep learning-based point source localization system (our previous work demonstrated providing these outputs directly to a robotic control system to track a needle tip using photoacoustic visual servoing^35^).

## 3 Results

### 3.1 Simulated Data Performance

**Fig. 3:**
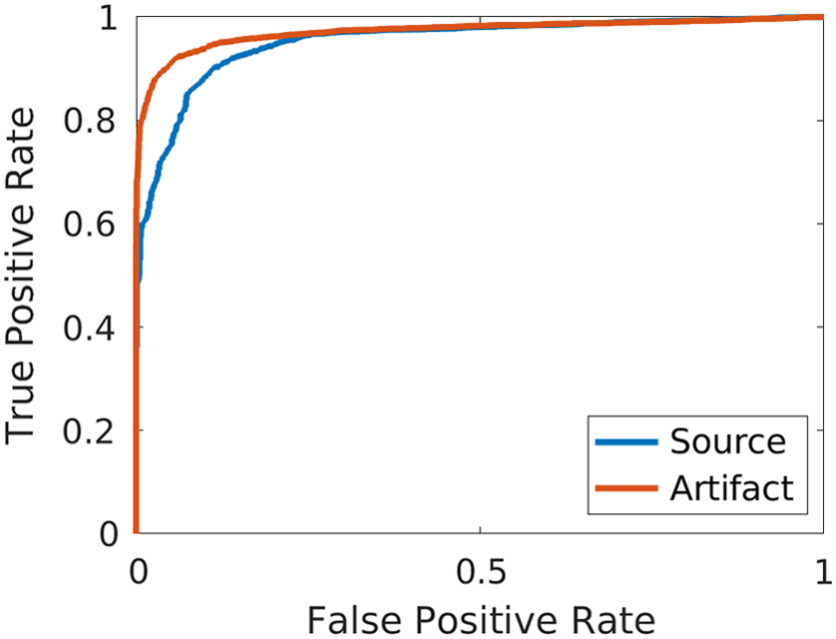
Receiver operating characteristic curves for the simulated source and artifact classes in the validation dataset.

Figure 3 shows ROC curves for simulated sources and artifacts in the validation dataset. These ROC curves reveal that the quality of detections was similar for both sources and artifacts, with AUC values of 0.953 and 0.972, respectively. Additional network performance metrics (i.e., recall, precision, F1 scores, misclassification rates, and missed detection rates) are reported in Table 2. While the network was better at detecting sources compared to reflection artifacts with recall values of 98.5% and 85.1% for sources and artifacts, respectively, the precision values were similarly high (i.e., 98.1% and 96.9%, respectively), resulting in F1 scores of 98.3% and 90.6% for sources and artifacts, respectively. The network was less susceptible to misclassification and missed detection errors for sources (i.e., 0.2% and 1.3%, respectively) compared to artifacts (i.e., 3.0% and 11.8%, respectively).

**Table 2:** Network performance on simulated sources and artifacts in the validation dataset.

Performance metric	Sources	Artifacts
Recall (%)	98.5	85.1
Precision (%)	98.1	96.9
F1 score (%)	98.3	90.6
Misclassification rate (%)	0.2	3.0
Missed detection rate (%)	1.3	11.8

**Fig. 4:**
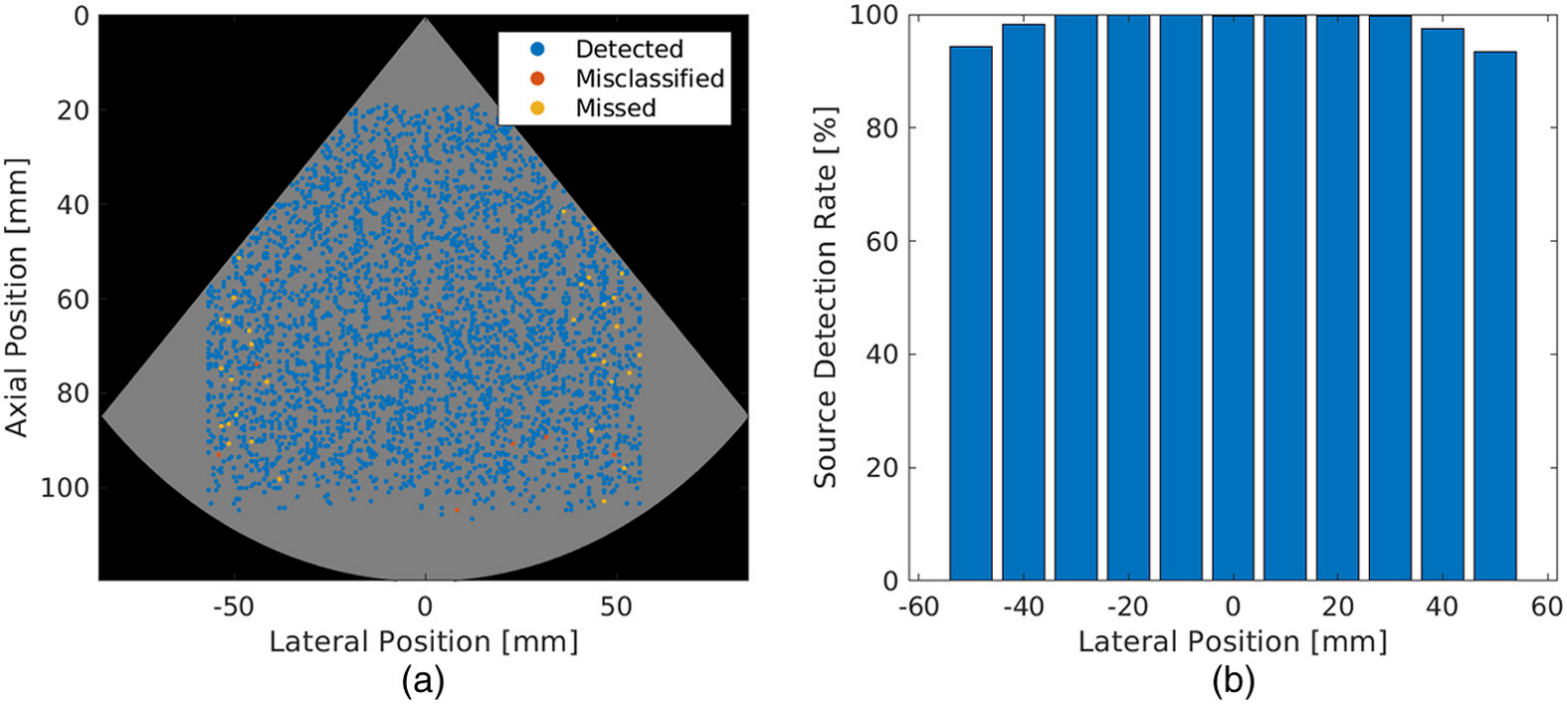
(a) Map of detected, misclassified, and missed sources in the simulated validation dataset overlaid on the scan-converted image FOV. (b) Source detection rates as a function of ground truth lateral positions relative to the transducer.

Figure 4 shows network performance as a function of ground truth source positions for the validation dataset. In Fig. 4(a), a map of correctly detected, misclassified, and missed sources are overlaid on a grid containing the FOV of the phased array transducer in gray. There is no apparent relationship between the axial position of the source and the detection, misclassification, and missed detection rates of the network. However, the source detection rate [shown in blue in Fig. 4(a)] appears to decrease with an increase in the lateral displacement of the source from the center of the transducer. Figure 4(a) also depicts an increase in missed sources that are laterally displaced by ±35  mm from the center of the transducer, as indicated by the increased presence of yellow circles near the edges of the transducer FOV. In addition, seven of the 4000 simulated sources in the validation dataset were misclassified as artifacts.

Confirming the qualitative observations described above, Fig. 4(b) shows a histogram of the source detection rate as a function of the lateral displacement of simulated sources from the center of the transducer. The network detected 99.7% of sources within ±5  mm of the transducer center. This detection rate was retained for sources with lateral displacements of up to ±30  mm from the transducer center. A decrease in source detection rate to 93.4% was observed as the lateral displacement increased to ±50  mm from the transducer center. Therefore, photoacoustic point source detection effectiveness is greatest near the center of the transducer, which is of most importance in photoacoustic visual servoing applications with deep learning.^35^

**Fig. 5:**
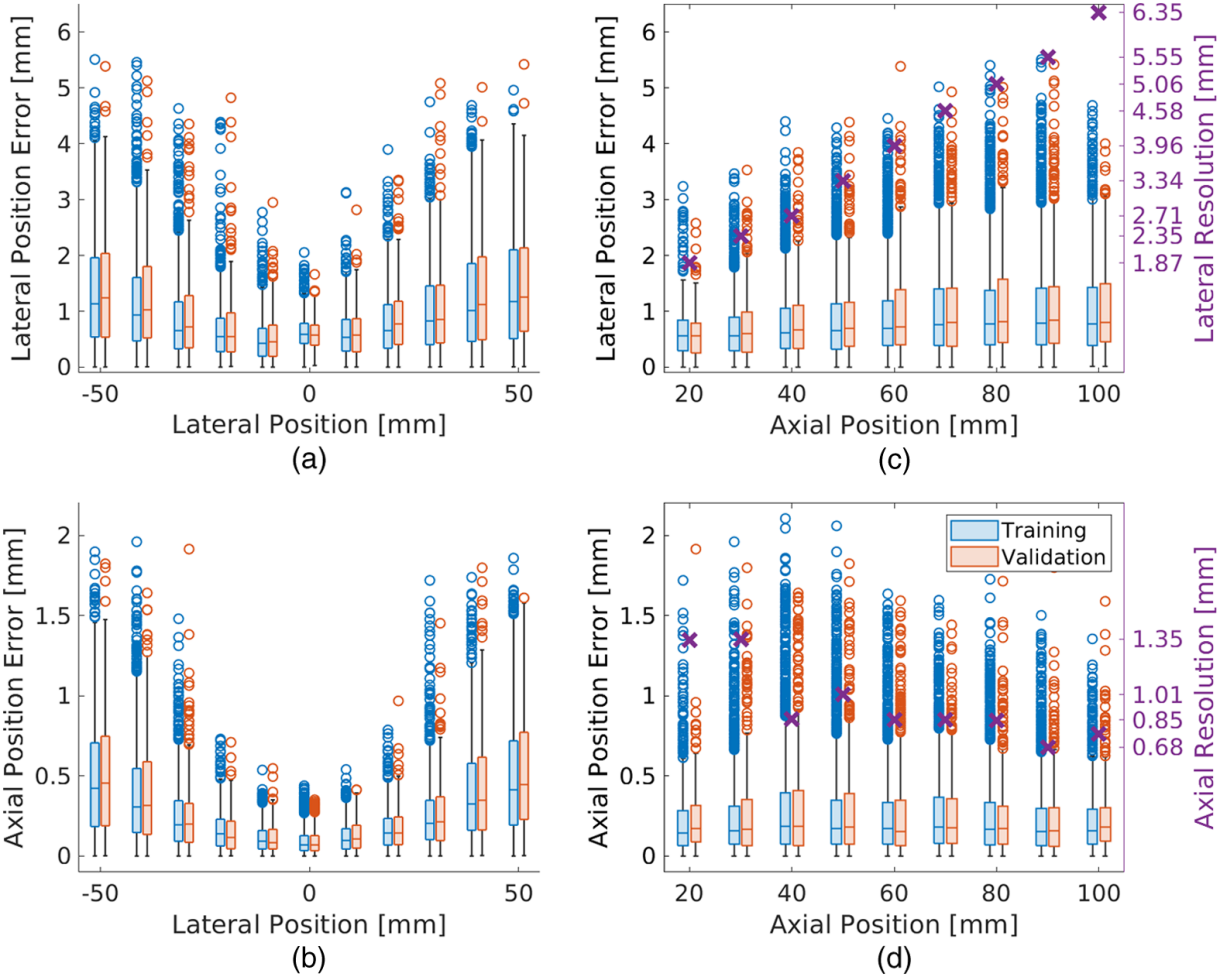
Absolute [(a), (b)] lateral and [(c), (d)] axial position errors of correctly identified sources as functions of the [(a), (c)] lateral and [(b), (d)] axial positions of the ground truth sources with respect to the ultrasound transducer in the simulated training and validation datasets. The mean (b) lateral and (d) axial resolutions reported in Table 3 are also shown for comparison (purple ×). The horizontal line within and the height of each box represent the median and interquartile range, respectively. The vertical lines above and below each box extend to the maximum and minimum values, excluding outliers (i.e., circles), which are defined as values exceeding 1.5 times the interquartile range.

Figure 5 shows box plots of the lateral and axial position errors of correctly identified sources as functions of lateral and axial ground truth positions relative to the transducer center for the simulated validation and training sets. The interquartile ranges and peak outlier magnitudes of both the lateral [Fig. 5(a)] and axial position errors [Fig. 5(c)] were lowest near the lateral center of the transducer, further highlighting the dependence on lateral positions noted above. In Fig. 5(b), an increase in interquartile range and peak outlier magnitudes was observed in the lateral position error as the depth increased from 20 to 100 mm. However, in Fig. 5(d), the axial position errors did not significantly change with variation in depth. In addition, position errors were generally larger in the lateral dimension [Figs. 5(a) and 5(b)] compared to the axial dimension [Figs. 5(c) and 5(d)]. Finally, the magnitudes of the median lateral and axial position errors were consistently smaller than the mean lateral and axial resolution, respectively, reported in Table 3 and shown in Figs. 5(b) and 5(d) for comparison. Note that the majority of position error magnitudes are less than the resolution of the ultrasound transducer. In the lateral dimension, 88.9% and 87.1% of network detections in the training and validation datasets, respectively, had absolute position errors less than the mean lateral resolution. In the axial dimension, 99.1% and 99.8% of detections in the training and validation datasets, respectively, had absolute position errors less than the mean axial resolution.

**Table 3:** Mean ± standard deviation of lateral and axial resolution measurements for the Alpinion SP1-5 phased array ultrasound transducer as functions of target depth (i.e., the axial position of the target) when the target was laterally centered (i.e., lateral position of 0 mm).

Axial position (mm)	Lateral resolution (mm)	Axial resolution (mm)
22.61	1.87±0.02	1.35±0.04
32.17	2.35±0.14	1.35±0.03
41.64	2.71±0.10	0.85±0.12
51.21	3.34±0.10	1.01±0.19
62.04	3.96±0.09	0.85±0.00
71.23	4.58±0.09	0.85±0.00
81.59	5.06±0.15	0.85±0.02
92.30	5.55±0.13	0.68±0.00
102.49	6.35±0.14	0.76±0.09

Table 4 reports the mean and standard deviation of the absolute lateral, absolute axial, and Euclidean distance errors in the simulated training and validation datasets. Similar mean absolute position errors were observed in the training and validation datasets in the lateral (i.e., 0.90 and 0.95 mm, respectively) and axial (i.e., 0.26 and 0.27 mm, respectively) dimensions. These mean absolute position errors were reduced for ground truth positions directly under the transducer compared to those outside the transducer aperture. These observations are similarly consistent when considering the Euclidean distance errors in Table 4 (i.e., similar errors for the training and validation datasets, decreased errors for ground truth positions directly under the transducer compared to outside the transducer).

**Table 4:** Mean ± standard deviation of absolute lateral, absolute axial, and Euclidean distance errors between network detections and ground truth simulated sources. Results are reported for all source positions and after stratifying by source lateral positions located between or within the zero-padded regions in processed channel data frames (i.e., under and outside the transducer, respectively).

		Lateral error (mm)	Axial error (mm)	Euclidean error (mm)
Training	All	0.90±0.78	0.26±0.28	0.96±0.79
	Under	0.55±0.33	0.10±0.08	0.57±0.32
	Outside	0.97±0.83	0.29±0.29	1.05±0.84
Validation	All	0.95±0.83	0.27±0.29	1.02±0.84
	Under	0.54±0.32	0.10±0.08	0.57±0.31
	Outside	1.04±0.87	0.30±0.31	1.12±0.89

### 3.2 Histogram Matching on *Ex Vivo* and *In Vivo* Heart Data

**Fig. 6:**
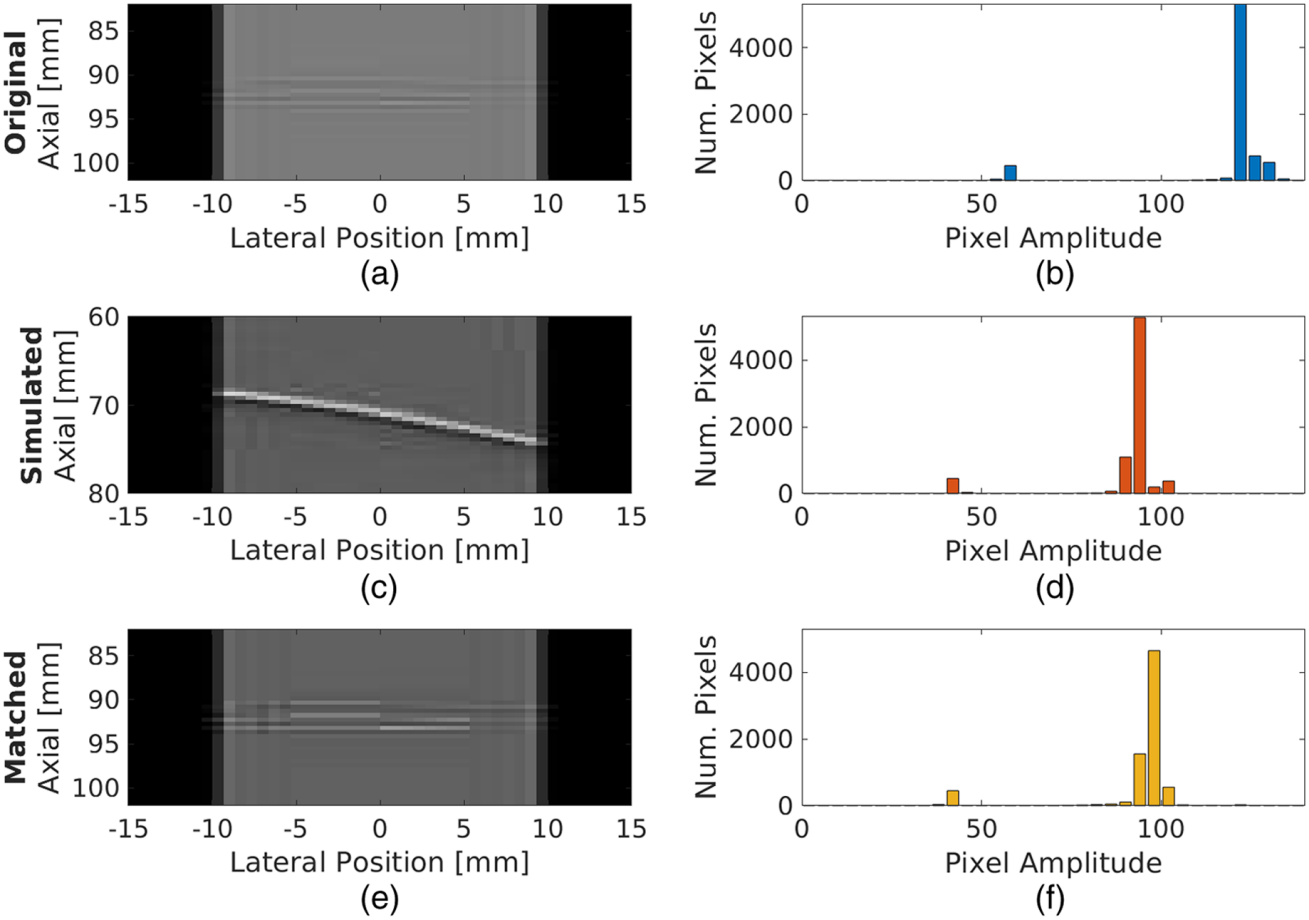
[(a), (c), (e)] Photoacoustic channel data frames and [(b), (d), (f)] corresponding histograms of amplitude data from images of a catheter tip in an *in vivo* swine heart from the *In Vivo* Heart 1 dataset [(a), (b)] before and [(e), (f)] after histogram matching with [(c), (d)] data from a simulated point source.

Figure 6 demonstrates the effect of the histogram matching procedure on a processed channel data frame from the *In Vivo* Heart 1 dataset. The wavefront corresponding to the catheter tip is initially difficult to identify [Fig. 6(a)], although the corresponding histogram indicates the presence of signals with two distinct amplitude ranges [Fig. 6(b)]. After histogram matching with a randomly selected simulated processed channel data frame [Figs. 6(c) and 6(d)], the visibility of the wavefront corresponding to the catheter tip is improved [Fig. 6(e)] as quantified by the improvement in ${\mathrm{gCNR}}_{\mathrm{ch}}$ from 0.182 before histogram matching to 0.796 after histogram matching. The corresponding amplitude histogram in Fig. 6(f) is more similar to the reference histogram in Fig. 6(d) when compared to the original *in vivo* histogram in Fig. 6(b). In this example, the TVD, JD, and $\chi^{2}$ statistics between the histograms of the channel data regions of the simulated and *in vivo* frames were successfully reduced from initial values of 0.989, −0.043, and 1.961, respectively, to values of 0.625, −0.842, and 0.926, respectively, for the histogram-matched result. Note that the zero-padded regions of each processed channel data frame were not included in this assessment because they remained unchanged after the histogram matching transformation.

Table 5 reports the mean and standard deviation of the TVD, JD, and $\chi^{2}$ statistics for histograms of the visible channel data regions of processed and histogram-matched channel data frames in the *ex vivo* and *in vivo* datasets with the corresponding simulated reference frames used for histogram matching. With histogram matching applied to the *Ex Vivo* Heart, *In Vivo* Heart 1, and *In Vivo* Heart 2 datasets, the mean TVD values decreased by 0.081, 0.082, and 0.048, respectively, the mean JD values decreased by 0.195, 0.198, and 0.147, respectively, and the mean $\chi^{2}$ statistics decreased by 0.237, 0.237, and 0.147, respectively. Overall, these results demonstrate the ability of histogram matching to reduce dissimilarities of *ex vivo* and *in vivo* data relative to the simulated data used to train the network.

**Table 5:** Mean ± one standard deviation of image amplitude histogram distances (i.e., TVD, JD, and $\chi^{2}$ statistic) between *ex vivo* and *in vivo* datasets and corresponding simulated channel data frames and the ${\mathrm{gCNR}}_{\mathrm{ch}}$ in *ex vivo* and *in vivo* processed channel data frames before and after histogram matching (HM).

Dataset		Relative to simulated data			${\mathrm{gCNR}}_{\mathrm{ch}}$
		TVD	JD	$\chi^{2}$ Statistic	
*Ex Vivo* Heart	Before HM	0.991±0.006	−0.027±0.016	1.972±0.018	0.606±0.265
	After HM	0.910±0.103	−0.222±0.221	1.735±0.291	0.640±0.280
*In Vivo* Heart 1	Before HM	0.990±0.004	−0.030±0.014	1.970±0.013	0.792±0.073
	After HM	0.908±0.081	−0.228±0.168	1.733±0.217	0.794±0.072
*In Vivo* Heart 2	Before HM	0.991±0.013	−0.021±0.023	1.976±0.032	0.348±0.125
	After HM	0.943±0.078	−0.148±0.179	1.829±0.225	0.386±0.129

**Fig. 7:**
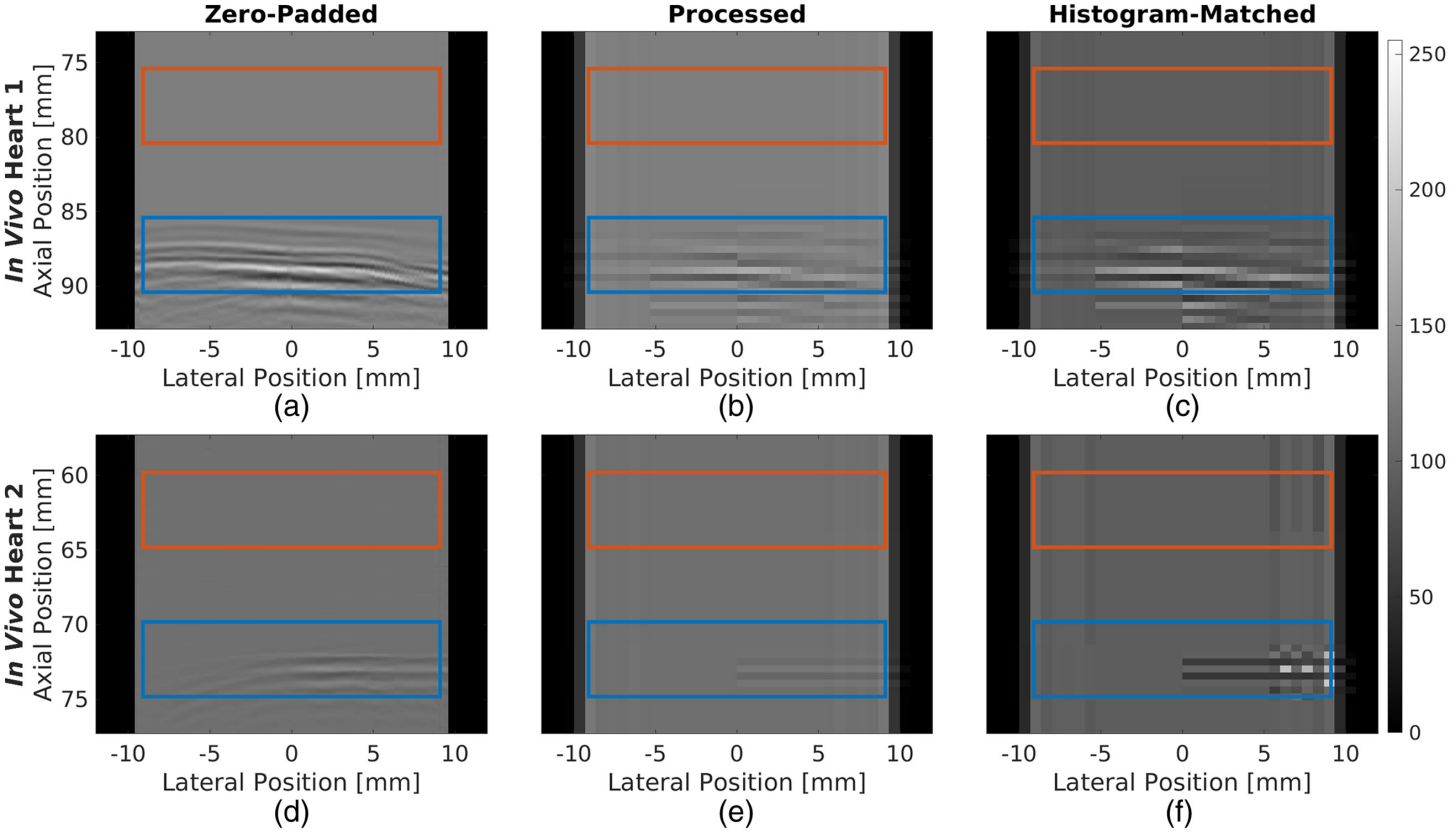
Example [(a), (d)] zero-padded, [(b), (e)] processed, and [(c), (f)] histogram-matched channel data frames from the *In Vivo* Heart 1 (top) and *In Vivo* Heart 2 (bottom) datasets, each originating from the same raw channel data. The ROIs correspond to the target (i.e., waveforms associated with the catheter tip) and background, defined to calculate the following ${\mathrm{gCNR}}_{\mathrm{ch}}$ measurements: (a) 0.935, (b) 0.753, (c) 0.854, (d) 0.597, (e) 0.221, and (f) 0.383.

Figure 7 shows examples of raw channel data images from the *In Vivo* Heart 1 and *In Vivo* Heart 2 datasets after zero-padding, resizing, and histogram matching, with the target and background ROIs shown with blue and orange boxes, respectively. For the *In Vivo* Heart 1 dataset, in Fig. 7(a), the waveform corresponding to the source spans the width of the channel data region in the zero-padded channel data frame. In addition, the waveform is visibly distinguishable from the background with a ${\mathrm{gCNR}}_{\mathrm{ch}}$ of 0.935. The detectability of this waveform was reduced after resizing [Fig. 7(b)], resulting in a ${\mathrm{gCNR}}_{\mathrm{ch}}$ measurement of 0.753. Despite this reduction, the network successfully identified the source in Fig. 7(b). After histogram matching [Fig. 7(c)], the detectability of the waveform improved with a measured ${\mathrm{gCNR}}_{\mathrm{ch}}$ of 0.854, and the network continued to successfully identify the source. In comparison, for the *In Vivo* Heart 2 dataset, after zero-padding [Fig. 7(d)], the signal amplitude of the waveform corresponding to the catheter tip was reduced when compared to that in Fig. 7(a), resulting in a reduced ${\mathrm{gCNR}}_{\mathrm{ch}}$ of 0.597. After resizing [Fig. 7(e)], the left edge of the waveform was indistinguishable from the background, with a ${\mathrm{gCNR}}_{\mathrm{ch}}$ of 0.221 (which is 0.376 lower compared to the zero-padded channel data frame), and the network did not detect this waveform. After histogram matching [Fig. 7(f)], the left edge of the waveform remained indistinguishable from the background, but the ${\mathrm{gCNR}}_{\mathrm{ch}}$ improved to 0.383, resulting in a successful detection of the source waveform. The last column of Table 5 summarizes the mean and standard deviation of ${\mathrm{gCNR}}_{\mathrm{ch}}$ measurements in processed channel data frames of the *ex vivo* and *in vivo* datasets before and after histogram matching. The mean ${\mathrm{gCNR}}_{\mathrm{ch}}$ measurements in the *Ex Vivo* Heart, *In Vivo* Heart 1, and *In Vivo* Heart 2 datasets demonstrate increases of 0.034, 0.002, and 0.038, respectively, with histogram matching. Overall, these results demonstrate the ability of our histogram matching technique to increase the detectability of the waveforms corresponding to the source in *ex vivo* and *in vivo* datasets.

Table 6 reports the precision, recall, F1 scores, misclassification rates, and missed detection rates for sources in the *ex vivo* and *in vivo* datasets, before and after histogram matching. No change was observed in the *In Vivo* Heart 1 dataset with recall, precision, and F1 scores of 100.0% and misclassification and missed detection rates of 0.0%, both before and after histogram matching. In the *Ex Vivo* Heart dataset, the recall, precision, and F1 scores increased by 7.8%, 1.4%, and 5.4%, respectively, after histogram matching, and the missed detection rate decreased by 8.2% after histogram matching. Conversely, the misclassification rate for the *Ex Vivo* Heart dataset increased from 0.0% to 0.4% after histogram matching. In the *In Vivo* Heart 2 dataset, the network output a single detection for the entire dataset of 40 images before histogram matching. This single detection was a true positive, resulting in a precision of 100.0% and a recall (i.e., detection rate) of 2.5%. After histogram matching, the number of network detections in the *In Vivo* Heart 2 dataset increased to 37 (35 of which were true positives). As a result, the precision decreased by 5.4% to 94.6% and recall increased by 85.0% to 87.5%, leading to improvements in the F1 score and missed detection rate, with no change to the misclassification rate.

**Table 6:** Network performance on *ex vivo* and *in vivo* data before and after histogram matching.

Performance metric	*Ex Vivo* Heart		*In Vivo* Heart 1		*In Vivo* Heart 2	
	Before	After	Before	After	Before	After
Recall (%)	71.2	79.0	100.0	100.0	2.5	87.5
Precision (%)	96.5	97.9	100.0	100.0	100.0	94.6
F1 score (%)	82.0	87.4	100.0	100.0	4.9	90.9
Misclassification rate (%)	0.0	0.4	0.0	0.0	0.0	0.0
Missed detection rate (%)	28.8	20.6	0.0	0.0	97.5	12.5

**Fig. 8:**
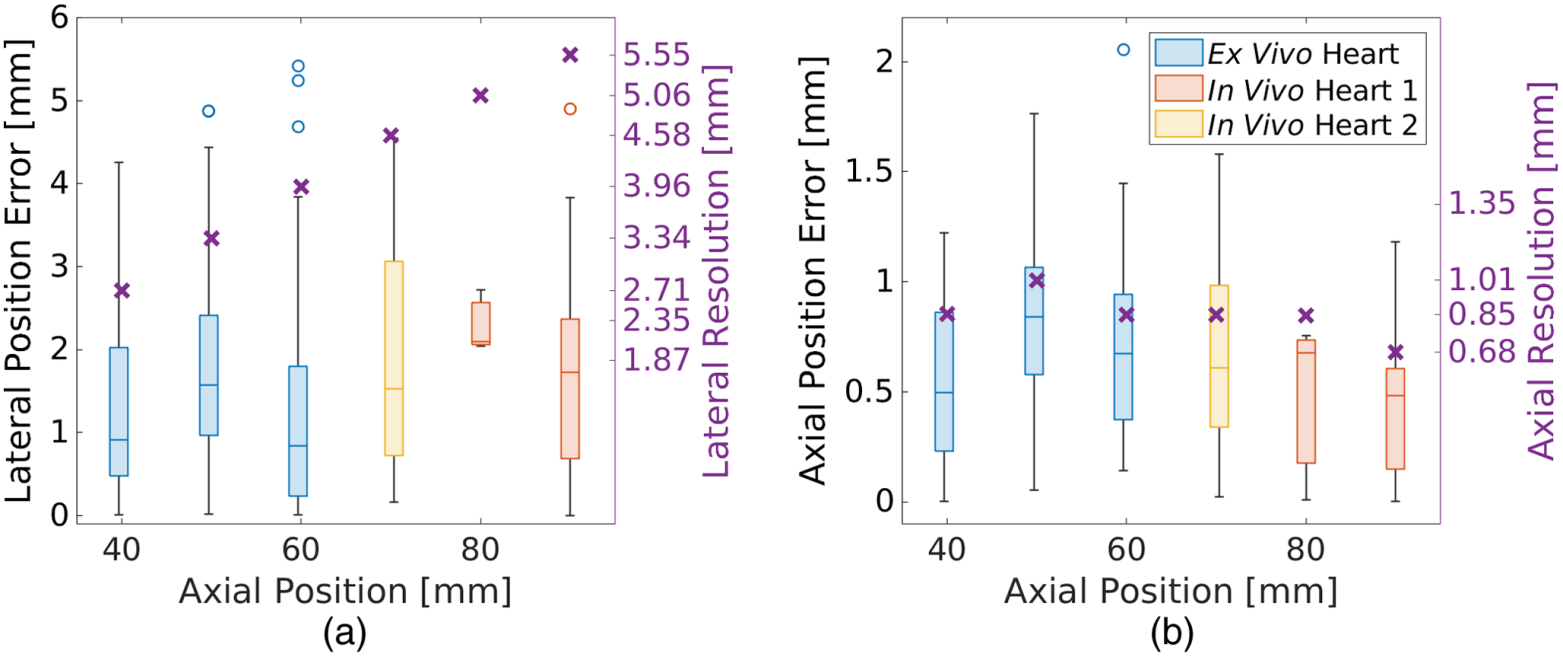
Absolute (a) lateral and (b) axial position errors of correctly identified sources as functions of the ground truth axial positions of the sources with respect to the ultrasound transducer in the *ex vivo* and *in vivo* datasets. The mean (a) lateral and (b) axial resolutions reported in Table 3 are also shown for comparison (purple ×). The horizontal line within and the height of each box represent the median and the interquartile range, respectively. The vertical lines extending above and below each box extend to the maximum and minimum values, excluding outliers (i.e., circles), which are defined as values exceeding 1.5 times the interquartile range.

Figure 8 shows the box plots of the lateral [Fig. 8(a)] and axial [Fig. 8(b)] position errors of correctly identified sources as functions of axial ground truth positions relative to the transducer for the *ex vivo* and *in vivo* datasets after histogram matching. Comparison of the position errors in Fig. 5 with the position errors in Figs. 8(b) and 8(d) reveals generally larger errors with the *ex vivo* and *in vivo* datasets (Fig. 8) relative to the simulated validation set results (Fig. 5) at similar axial target depths, though the outliers in the simulated dataset are more consistent with the *ex vivo* and *in vivo* results. In addition, comparison of the position errors in Fig. 8 with the corresponding resolution measurements reveals that the median position errors are consistently lower than the corresponding resolution measurements for each source depth. When compared to the lateral and axial resolution reported in Table 3 (with means replicated in Fig. 8), the majority of position error magnitudes are smaller than the resolution (similar to the results obtained with the simulated datasets in Fig. 5). In the *Ex Vivo* Heart, *In Vivo* Heart 1, and *In Vivo* Heart 2 datasets, 88.6%, 100.0%, and 100.0% of network detections, respectively, had absolute lateral errors less than the mean lateral resolution. In the axial dimension, 67.2%, 86.7%, and 62.9% of network detections in the *Ex Vivo* Heart, *In Vivo* Heart 1, and *In Vivo* Heart 2 datasets, respectively, had absolute axial position errors less than the mean axial resolution of the ultrasound transducer.

### 3.3 Deep Learning-Based Improvement in Source Visualization

**Fig. 9:**
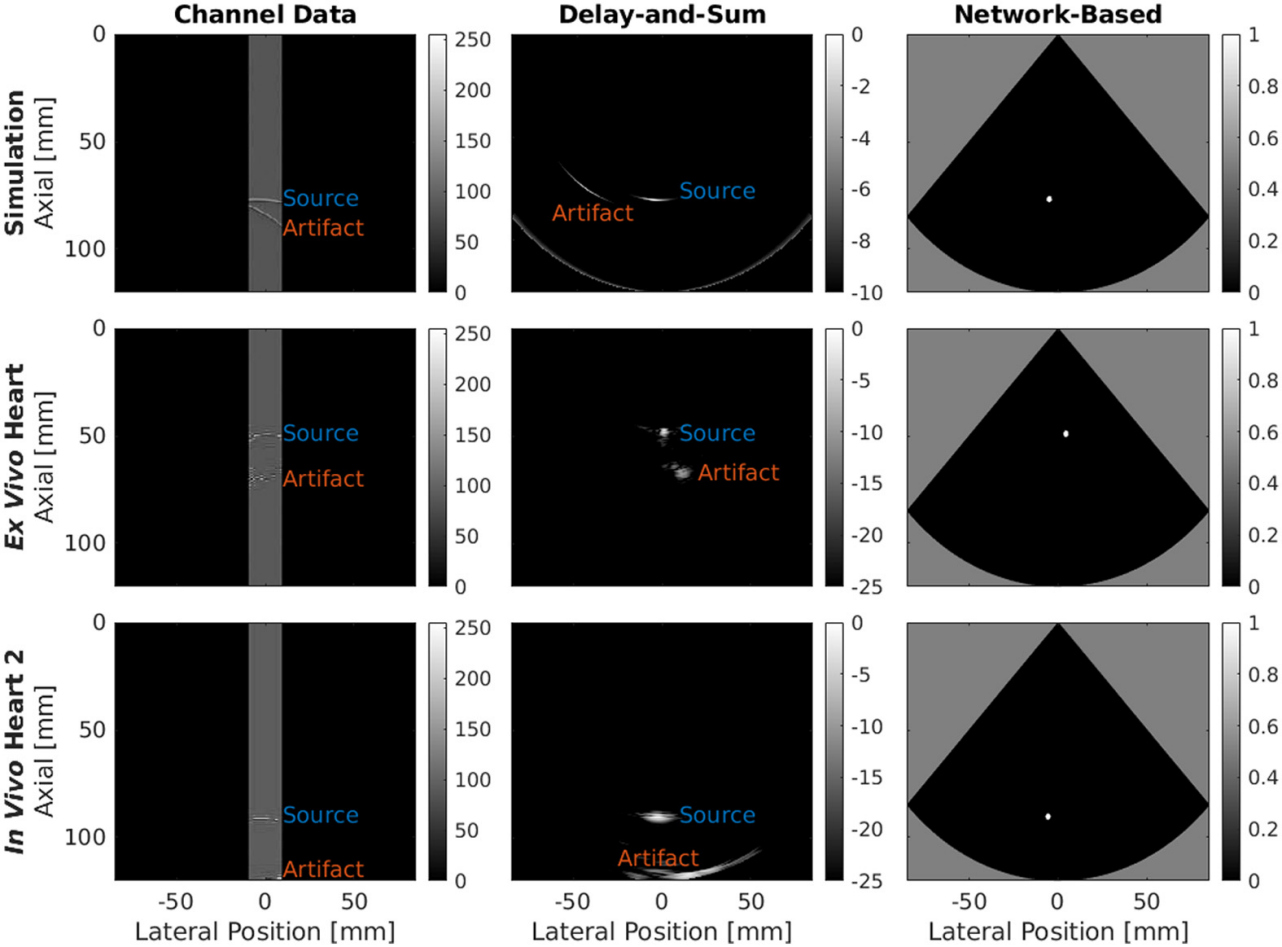
Simulated, *ex vivo* swine heart, and *in vivo* swine heart (top, middle, and bottom, respectively) samples of raw photoacoustic channel data, DAS images, and convolutional neural network-based images (left, center, and right, respectively) obtained with a phased array transducer.

Figure 9 shows zero-padded channel data, DAS-beamformed images, and network-based images visualizing photoacoustic point sources in simulated, *ex vivo*, and *in vivo* data (from the simulated validation, *Ex Vivo* Heart, and *In Vivo* Heart 2 datasets, respectively). The zero-padded channel data show waveforms corresponding to sources and artifacts spanning the width of the raw channel data region. In addition, the channel data regions of the *ex vivo* and *in vivo* images show distortions in the waveforms in the axial dimension. The DAS-beamformed images show distortions in the source shapes with the energy from the point source dispersed over a wider region in the DAS images compared to the network-based images. The DAS-beamformed images also contain reflection artifacts, resulting in potential confusion regarding the location of the point source. This limitation is overcome in the network-based images. In each case, the network-based image provides the clearest view of the source as a white circle on the black background denoting the image FOV. The radius of each circle is 2σ=1.68  mm, based on the standard deviation of the Euclidean distance error reported in Table 4 for the validation dataset (consisting of all data combined).

## 4 Discussion

This paper is the first to present deep learning-based photoacoustic source localization results achieved within *ex vivo* and *in vivo* hearts with a phased array transducer. To successfully detect point sources and reflection artifacts at any location in the phased array FOV, we introduce new methods to prepare the raw channel data frames for input to the deep neural network. These methods comprise a novel combination of: (1) zero-padding channel data frames to match the FOV of a scan-converted image, (2) resizing the zero-padded images to improve the network performance, and (3) histogram matching *ex vivo* and *in vivo* images to simulated images from a validation dataset. Neither zero-padding nor the associated extrapolation of waveform peaks enabled by zero-padding were applied to previous linear array data or networks.^28^^–^^32^^,^^57^

We can appreciate that zero-padding and image resizing contributed to the high network performance in simulated data (i.e., recall and precision of 98.5% and 98.1%, respectively, see Table 2) based on the following observations. First, the extrapolation of source positions from partially visible waveforms, which would not have been possible without zero-padding (due to limitations surrounding the placement of bounding boxes), indicates that zero-padding was a major contributing factor to the performance achieved by our network (Table 2). Second, the recall and precision values reported in Table 2 exceeded the recall and precision values of 84.3% and 90.7%, repectively, which were previously achieved by Allman et al.^33^^,^^34^ with zero-padding of phased array data. This performance improvement is likely due to the additional image resizing step that we implemented for the first time herein. Otherwise, with partially visible waveform peaks, our performance was higher than that achieved by Allman et al.^31^ with linear array data and fully visible waveforms (i.e., recall and precision values of 91.6% and 89.4%, respectively), which is likely due to multiple domain differences.

The inclusion of histogram matching and corresponding ${\mathrm{gCNR}}_{\mathrm{ch}}$ improvements (Table 5) ultimately resulted in recall improvements of 7.8% and 85.0% relative to the pre-histogram-matching performance on the *Ex Vivo* Heart and *In Vivo* Heart 2 datasets, respectively (Table 6). There were also some metrics that were not impacted or improved by histogram matching, revealing three key insights regarding its implementation. First, it is important to consider the effects of histogram matching on both recall and precision (i.e., the F1 score) when attempting to improve network performance, as an increase in one metric may be accompanied by a decrease in the other. Second, acceptably low misclassification rates were achieved before and after histogram matching, suggesting that signal amplitude is not the only factor considered by the network for the classification task. Third, histogram matching did not impact the already excellent network performance (e.g., 100.0% recall, precision, and F1 scores) on the *In Vivo* Heart 1 dataset, indicating that the approach will not degrade otherwise excellent performance.

The objective of histogram matching is to reduce signal amplitude dissimilarities between simulated and experimental training and testing data, respectively. However, even though there was an initial amplitude dissimilarity between the simulated and *In Vivo* Heart 1 datasets (Fig. 6), reducing this dissimilarity with histogram matching did not affect the already excellent network performance. Thus, dissimilarities are evidently not the only factor affecting the performance of a simulation-trained network when applied to *ex vivo* and *in vivo* datasets. Other possible factors include the absolute signal amplitudes in the *ex vivo* and *in vivo* datasets, the shapes of the waveforms corresponding to the sources, and the position distributions of sources relative to the transducer.^33^^,^^34^ In addition, the performance significantly improved when applying histogram matching to data acquired with low laser energies (i.e., 608.5  μJ for *In Vivo* Heart 2 versus 2.67 mJ for *In Vivo* Heart 1 datasets), which highlights the potential of our techniques to reduce the minimum laser energy required to ensure consistent point source detection and localization (e.g., to achieve system miniaturization during photoacoustic-guided surgical and interventional procedures^56^ and safe imaging under extended procedure durations^59^).

As opposed to previous results with linear array networks, which achieved similar localization errors across multiple depth or lateral positions,^32^ the position errors achieved by the network and approach presented herein depended on the ground truth lateral and axial positions, as shown in Figs. 5(a)–5(c). The increases in lateral and axial position errors with lateral position [Figs. 5(a) and 5(c), respectively] for the phased array network are likely due to the required extrapolation of the position of the waveform peak. In particular, waveform information decreases with the lateral displacement of the source from the transducer center, yet knowledge of the waveform peak is critical to accurately detect and locate sources. In addition, the larger lateral position errors with increasing depth [Fig. 5(b)] are likely due to the increased range of lateral positions with depth arising from the geometry of a phased array image FOV.

The mean absolute axial and lateral position errors for simulated sources in the validation dataset were 0.27 and 0.95 mm, respectively, as reported in Table 4. These errors are larger than the 0.088 mm (axial) and 0.103 mm (lateral) position errors reported by Bell^57^ when summarizing previous work with linear array networks, likely because of the resolution difference between the phased and linear array transducers. For simulated sources in the validation dataset, the phased array network presented herein achieved mean absolute axial position errors ranging 0.10 to 0.30 mm, depending on the ground truth source position, as reported in Table 4. A majority (i.e., 99.8%) of these axial errors are within the mean axial resolution measurements of the phased array transducer [Fig. 5(d)]. Similarly, a majority of the obtained lateral errors in Fig. 5(b) (i.e., 87.1%) are within the mean lateral resolution measurements in Fig. 5(b) for corresponding target depths. It is promising that a majority of these results are within the resolution of the transducer.

Despite the nuances described above, the simulation-trained network presented herein successfully translated to experimental *ex vivo* and *in vivo* data, in some cases with better performance on *ex vivo* and *in vivo* data than on the simulated validation data. As reported in Tables 2 and 6, the ranges of recall (i.e., 79.0% to 100.0%), precision (i.e., 94.6% to 100.0%), and F1 scores (i.e., 87.4% to 100.0%) achieved by our network on *ex vivo* and *in vivo* data were consistent with values achieved for the simulated validation dataset. In addition, our network achieved higher recall, precision, and F1 scores in the *In Vivo* Heart 1 dataset (i.e., 100.0%, 100.0%, and 100.0%, respectively) compared to the simulated validation dataset (i.e., 98.5%, 98.1%, and 98.3%, respectively). These improved performance values are likely due to the reduced lateral displacements of the source from the transducer in the *ex vivo* and *in vivo* data compared to the simulated data, leading to improved source detection rates. The higher energies employed to acquire this dataset could also be responsible for the improved performance.

One limitation of our approach is that catheter tips outside the imaging plane of the transducer may not be detected if located outside of the depth-dependent elevation beamwidth (i.e., at least 2 mm width based on data provided by the manufacturer). However, when not in the heart, the catheter is anticipated to be confined to vessels, which will have a diameter no larger than 2.2 cm.^60^^–^^65^ In addition, with the lateral dimension of the transducer aligned with the direction of catheter travel, we previously demonstrated the successful implementation of a real-time, robot-assisted photoacoustic target tracking system using phased array ultrasound transducers that provide 2D images.^20^^,^^35^ These systems compensated for the reported elevation resolution by using the robotic control and elevation plane search algorithms developed by our group.^17^^,^^20^^,^^35^ Alternatively, a transducer with volumetric imaging capabilities can be employed to localize the catheter tip in three spatial dimensions in a single image. Regarding reflection artifact detection, our study was limited to characterizing this particular performance on simulated data, because characterizations on *ex vivo* or *in vivo* data would have required manual annotations derived from photoacoustic images (e.g., the DAS-beamformed image of the *In Vivo* Heart 2 dataset in Fig. 9), which is not always feasible (e.g., due to uncertainty about the peak locations of partially visible waveforms in phased array channel data).

The proposed network-based photoacoustic source visualization method for phased array data has potential utility in multiple possible future scenarios. First, as previously proposed by Allman et al.,^31^^,^^34^ this method may be used to distinguish between photoacoustic point sources and reflection artifacts, relying on the classification accuracy of the network and avoiding the inaccuracies inherent to traditional image reconstruction algorithms using beamforming. Second, this method may be integrated with our previously presented deep learning-based photoacoustic visual servoing system,^35^ leveraging the network outputs generated for robotic tracking to simultaneously generate high quality human-interpretable images of the source being tracked. Third, these network-based images may be superimposed on traditional ultrasound images to provide clinicians with real-time visual information of catheter tips during cardiac procedures. Finally, the proposed methods have the potential to be extended to other applications of deep learning in photoacoustics^66^ and biomedical optics.^67^^,^^68^

## 5 Conclusion

We successfully demonstrated new approaches to improve the performance of a deep learning-based photoacoustic point source localization system operating on raw channel data acquired with a phased array transducer for cardiac applications. Image resizing in tandem with channel data zero-padding was implemented during network training to detect and localize point sources in simulated data. We successfully translated this simulation-trained network to *ex vivo* and *in vivo* images of a catheter tip. We characterized the performance of the network on this experimental data before and after introducing a novel amplitude-based histogram matching strategy. Subsequently, we demonstrated the applicability of our successfully trained network to improve the visibility of photoacoustic point sources and remove reflection artifacts in phased array photoacoustic images. Promising applications of this work include integration with previously presented deep learning-based robotic visual servoing systems leveraging existing network outputs to ultimately improve robotic tracking and human-interpretable visualization of catheter tips during cardiac procedures.

## Code, Data, and Materials Availability

Part of the code used to train, validate, and test the neural network was obtained from https://github.com/facebookresearch/detectron2 and is publicly available. The remainder of the code and data used in this study can be made available upon reasonable request to the corresponding (MRG) and senior (MALB) authors.
